# Successful treatment of recalcitrant Hailey-Hailey disease with ixekizumab

**DOI:** 10.1016/j.jdcr.2026.04.055

**Published:** 2026-05-05

**Authors:** Garrett Bohrnstedt, Kaleb Bohrnstedt, Westley Carter

**Affiliations:** Department of Dermatology, A.T. Still University/Northeast Regional Medical Center, Kirksville, Missouri

**Keywords:** familial benign pemphigus, Hailey-Hailey disease, IL-17 inhibitor, ixekizumab

## Introduction

Hailey–Hailey disease (HHD) is an autosomal dominant genodermatosis caused by mutations in *ATP2C1*, resulting in calcium homeostasis and defective keratinocyte adhesion. Clinically, it presents with recurrent vesicles, erosions, and fissured plaques, most commonly affecting intertriginous and friction-prone areas.^1^ The management remains challenging, and many patients experience recurrent flares despite topical therapies, systemic antibiotics, and immunomodulatory agents. We describe a case of HHD refractory to multiple agents that was successfully treated with Ixekizumab, an anti-interleukin (IL)-17A monoclonal antibody.

## Case report

A 71-year-old woman presented with a decades-long history of a blistering and erosive dermatosis involving intertriginous and friction-associated areas. The condition had been clinically diagnosed in early adulthood as Hailey–Hailey disease. There was no known family history of Hailey–Hailey disease. The condition was characterized by recurrent flares of painful, erythematous, fissured, and crusted plaques involving the neck, chest, abdomen, back, groin, and extremities. Short courses of systemic corticosteroids provided the most consistent improvement; however, disease rapidly recurred upon tapering.

Prior treatments included topical corticosteroids, topical calcineurin inhibitors, topical antibacterials, antiseptic washes, multiple courses of systemic antibiotics, naltrexone 3 mg BID, dupilumab for 6-8 m, and nemolizumab for approximately 3 months. Dupilumab improved pruritus but resulted in minimal improvement of cutaneous disease, while nemolizumab was ineffective and subjectively worsened symptoms.

Physical examination revealed widespread circinate to serpiginous groupings of eroded and crusted vesicles on an erythematous base, involving a majority of the trunk and extremities. Additional psoriasiform plaques with silvery scale were present on the proximal thighs. Disease severity was substantial, with an estimated body surface area involvement of 57%, a PASI score of 26.4, and pruritus severity rated as 9/10. The PASI scoring system was utilized to quantify severity due to the psoriasiform morphology of several lesions; however, the patient was not diagnosed with concomitant psoriasis.

**Fig 1:**
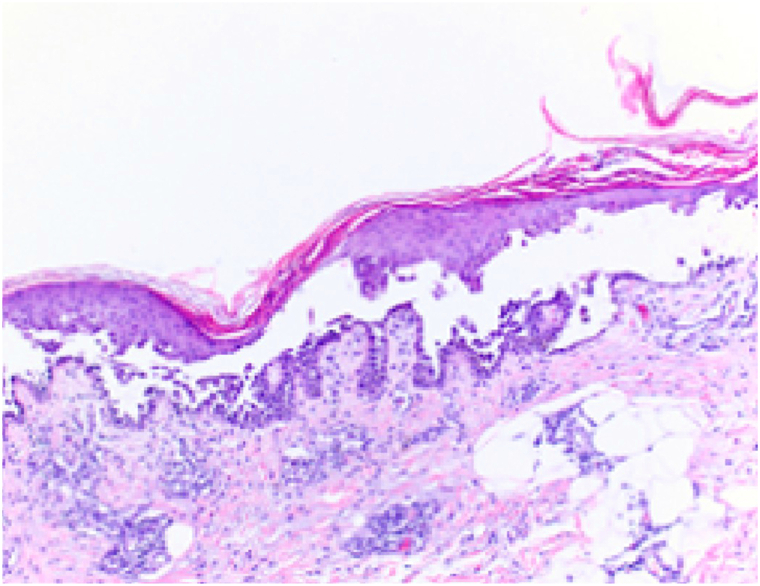
Hailey–Hailey disease. Histopathologic examination of a punch biopsy from the left lateral thigh demonstrating suprabasal acantholysis with prominent background inflammation, consistent with Hailey–Hailey disease with a superimposed hypersensitivity reaction.

Punch biopsies from representative lesions on the left lateral thigh and right lumbar back demonstrated histopathologic findings consistent with Hailey–Hailey disease, including suprabasal acantholysis (Fig 1). Prominent background inflammation suggested a superimposed hypersensitivity reaction. Given the severity, refractoriness, and psoriasiform features, a therapeutic pivot was made to IL-17A inhibition with ixekizumab.

Ixekizumab was initiated using standard loading dosing. Within 2 weeks, the patient reported marked improvement in pain and pruritus, with visible reduction in erosions and vesiculation. By week 4, pruritus had fully resolved, systemic corticosteroids were discontinued, and topical therapies were no longer required. Continued improvement was observed with subsequent injections.

**Fig 2:**
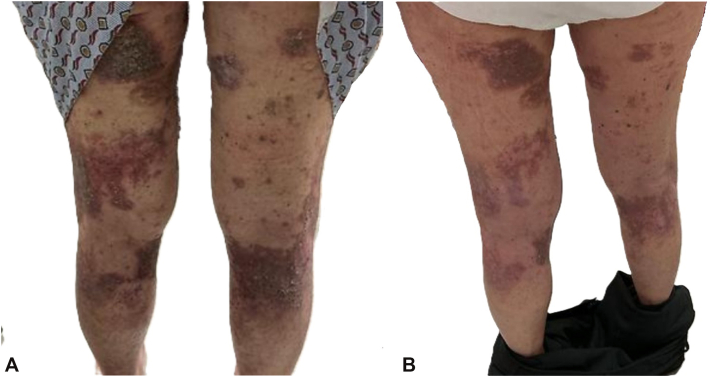
Hailey–Hailey disease. Posterior view of the lower extremities demonstrating **(A)** widespread erythematous, crusted, and eroded plaques with psoriasiform features prior to treatment, and **(B)** marked clinical improvement with residual post-inflammatory hyperpigmentation following ixekizumab therapy.

Objective disease metrics demonstrated progressive improvement: body surface area involvement decreased from 57% at baseline to 17% by week 4 and to 5% by week 8. PASI improved from 26.4 at baseline to 3.6 by week 8, with complete resolution of pruritus. Residual findings were limited to post-inflammatory scarring and minimal hypertrophic plaques. The patient tolerated therapy without infections or candidiasis (Fig 2).

## Discussion

This case demonstrates a rapid, durable, and steroid-sparing response of severe, biopsy-confirmed Hailey–Hailey disease (HHD) to IL-17A inhibition after failure of multiple conventional and biologic therapies. HHD is classically regarded as a disorder of impaired keratinocyte adhesion resulting from abnormal processing and trafficking of junctional proteins, leading to epidermal acantholysis.^1^ However, recurrent barrier disruption and chronic erosions suggest that secondary inflammatory pathways may contribute to ongoing disease activity.^2^^,^^3^

Targeted inhibition of IL-17A represents a biologically plausible therapeutic approach in this setting. A recent immunohistochemical study of 34 patients with acantholytic and blistering genodermatoses, including HHD, demonstrated a 5- to 7-fold increase in IL-17A–positive cells in lesional skin compared with normal controls. This finding was supported by increased expression of downstream S100 calcium-binding proteins (S100A7, S100A8, and S100A9), indicating activation of the IL-17 inflammatory axis.^4^

Together, these data support a contributory role for IL-17A–mediated inflammation in HHD pathogenesis. Although the disease originates from ATP2C1-associated calcium dysregulation and impaired keratinocyte adhesion, repeated epidermal injury may perpetuate a chronic inflammatory environment in which IL-17A amplifies keratinocyte stress responses, neutrophil recruitment, and microbial-driven inflammation. IL-17A pathway inhibition therefore offers a targeted, mechanism-based treatment strategy rather than nonspecific immunosuppression.

There is limited literature regarding the use of IL-17A antagonists in HHD. This case expands the current therapeutic landscape by demonstrating that ixekizumab can induce rapid and sustained remission in severe, treatment-refractory disease. While further studies are needed to define the role of IL-17A inhibition in HHD management, this report highlights its potential as a steroid-sparing option in selected patients.

### Declaration of generative AI and AI-assisted technologies in the writing process

During the preparation of this work the author(s) used ChatGPT to assist with drafting and refining the written text, and ensure compliance with formatting guidelines. After using this tool/service, the author(s) reviewed and edited the content as needed and take(s) full responsibility for the content of the publication.

## Conflicts of interest

None disclosed.

## References

[1] Kodali N., Lyons A., Shiver M. (2024). Hailey-Hailey disease: a review and an approach to management. JAAD Int.

[2] Echeandia-Francis C., Alzawahra W., Strachan D. (2024). Efficacy of interleukin-12/23 and interleukin-23 inhibitors in Hailey-Hailey disease. JAAD Case Rep.

[3] Chen A., Doan L. (2023). Adalimumab therapy used successfully for recalcitrant Hailey-Hailey disease. JAAD Case Rep.

[4] Javid A.H., Li D., Technau-Hafsi K., Has C. (2023). Interleukin-17A immune pattern across genetic acantholytic and blistering disorders. Clin Exp Dermatol.

